# Favorable Outcome after Liver Transplantation in an Infant with Liver Failure Due to Deoxyguanosine Kinase Deficiency

**DOI:** 10.3390/jcm13185356

**Published:** 2024-09-10

**Authors:** Alina Grama, Gabriel Benţa, Alexandru Stefan Niculae, Alexandra Mititelu, Claudia Simu, Otilia Fufezan, Xavier Stephenne, Raymond Reding, Catherine de Magnee, Roberto Tambucci, Etienne Sokal, Tudor Lucian Pop

**Affiliations:** 1 2nd Pediatric Discipline, Mother and Child Department, “Iuliu Haţieganu” University of Medicine and Pharmacy, 400012 Cluj-Napoca, Romania; gabi.benta@gmail.com (G.B.); niculae.alexandru.stefan@elearn.umfcluj.ro (A.S.N.); alexandra.mititelu@elearn.umfcluj.ro (A.M.); sirbe.claudia.luminita@elearn.umfcluj.ro (C.S.); 2 Centre of Expertise in Pediatric Liver Rare Disorders, 2nd Pediatric Clinic, Emergency Clinical Hospital for Children, 400177 Cluj-Napoca, Romania; 3 Imaging Department, Emergency Clinical Hospital for Children, 400370 Cluj-Napoca, Romania; otilia.fufezan@gmail.com; 4 Cliniques Universitaires Saint Luc, Université Catholique de Louvain, 1200 Bruxelles, Belgium; xavier.stephenne@uclouvain.be (X.S.); raymond.reding@uclouvain.be (R.R.); catherine.demagnee@uclouvain.be (C.d.M.); roberto.tambucci@uclouvain.be (R.T.); etienne.sokal@uclouvain.be (E.S.)

**Keywords:** deoxyguanosine kinase deficiency, children, mitochondrial disorder, liver transplantation, outcome

## Abstract

**Introduction**: Deoxyguanosine Kinase (DGUOK) deficiency is a very rare disorder characterized by liver dysfunction, neurological manifestations, and metabolic disorders secondary to severely reduced mitochondrial DNA content. These patients develop early-onset liver failure, and their liver transplantation (LT) indication remains debatable due to the possibility of neurological involvement. **Case Report**: We present the case of a 6-month-old female diagnosed with DGUOK deficiency who developed liver failure. At 9 months, she underwent a living-related LT with an initial favorable evolution under immunosuppression therapy with tacrolimus. Four months after LT, she presented two prolonged bacterial and Rotavirus enteritis episodes. She developed classical post-transplant complications (severe renal tubular acidosis type IV, secondary to the high tacrolimus level, and post-transplant lymphoproliferative disease) during these episodes. Her condition deteriorated progressively, with reversible hypotonia and significant weight loss. However, the neurological evaluation did not reveal any signs suggestive of the progression of the underlying disease. A few months later, her clinical features and laboratory parameters improved considerably. **Conclusions**: This case highlights the unpredictable evolution of children with LT for liver failure due to DGUOK deficiency.

## 1. Introduction

Mitochondrial DNA (mtDNA) depletion syndromes (MDS) are a group of autosomal recessive disorders expressed by a reduced mtDNA copy number that leads to the deficient synthesis of respiratory chain complexes I–V and perturbed energy metabolism. The liver, brain, heart, kidney, and skeletal muscle are the most energy-dependent tissues of the body, and they are at risk of mtDNA depletion [1].

Mitochondrial deoxyguanosine kinase (DGUOK), encoded by the DGUOK gene, converts deoxyguanosine and deoxyadenosine to deoxyguanosine monophosphate and deoxyadenosine monophosphate. DGUOK and thymidine kinase (TK) are mitochondrial matrix proteins that provide deoxyribonucleotides for the synthesis of mtDNA. These proteins play an essential role in maintaining mtDNA and preserving the normal function of the respiratory chain complexes (I, III, IV, V) [2]. Variants in DGUOK or TK result in the reduced synthesis of mitochondrial deoxyribonucleotide triphosphate (dNTP), the main ingredient for mtDNA, leading to mtDNA depletion [3].

Depending on their genetic background, MDS are highly variable and may affect one organ or a combination of organs. Regarding their clinical presentation, MDS may present as the hepato-cerebral form (variants in the DGUOK, MPV17, POLG, or C10orf2 genes), myopathic form (variants in the TK2 gene), encephalomyopathic form (variants in the SUCLA2, SUCLG1, or RRM2B genes), or neurogastrointestinal form (variants in the TYMP gene) [3]. 

DGUOK deficiency is a very rare disorder characterized by liver dysfunction (jaundice, cholestasis, hepatomegaly, and elevated transaminases), neurological manifestations (hypotonia, nystagmus, opsoclonus, and psychomotor retardation), and metabolic disorders (lactic acidosis or hypoglycemia) secondary to severely reduced mtDNA. 

This report aims to present a case of DGUOK deficiency with a complex clinical evolution before and after liver transplantation (LT) and a good prognosis so far.

## 2. Case Report

We report the case of a 6-month-old female diagnosed with DGUOK deficiency who developed acute liver failure (ALF). The girl was referred to our hospital with a diagnosis of neonatal cholestasis. She was born at 39 weeks of gestation, by cesarean section for the maternal indication (uterine scar), with a weight of 3000 g, a length of 50 cm, and normal neonatal adaptation. The pregnancy was uneventful, and she was periodically monitored at a local hospital. There was no consanguinity, and her family has no history of inherited disease. The patient’s medical history mentions early-onset neonatal sepsis treated with ampicillin and gentamicin. 

She presented with jaundice and moderate hepatomegaly (liver at 3 cm); exhibited normal development for her age, with a weight 6 kg, a height of 63 cm, and a weight for length z-score of −1.1 (percentile 13.8); and had no neurological defects and no sign of encephalopathy. The initial laboratory parameters in our unit revealed increased transaminases (aspartate aminotransferase, AST, 148 U/L and alanine aminotransferase, ALT, 86 U/L), high bilirubin levels (total bilirubin 4.32 mg/dL, conjugated bilirubin 2.51 mg/dL) and cholestasis (increased gamma-glutamyl transferase, GGT, 186 U/L), normal albuminemia (4.5 g/dL), normal glycemia (75 mg/dL), and prolonged prothrombin time (20 s) with INR 1.5. Her creatine kinase and metabolic profile were normal. After eliminating other classical and curable etiologies of neonatal cholestasis, we performed whole exome analysis using the next-generation sequencing (NGS) technique (Illumina, San Diego, CA, USA HiSeq PE150, Human WES 50X per sample = 6G per sample, Agilent Technologies, Santa Clara, CA, USA SureSelect Human All Exon V6, Genome hg38annotated variants using ANNOVAR, version 20191024). The results confirmed the diagnosis of DGUOK deficiency (homozygous status for c.G3A: p.M1I variant, considered as a null variant). 

During the following months, the patient’s evolution was unfavorable. At 9 months, she developed ALF (severe coagulopathy with bleeding at the sites of venous puncture, and INR 6.93 not corrected with vitamin K), ascites with hypoalbuminemia 2.8 g/dL, severe hypoglycemia (18 mg/dL), and lactic acidosis (9 mmol/L). She was treated with high concentrations of glucose (10–12 mg/kg/min), fresh frozen plasma (FFP), prothrombin complex concentrate (PCC), recombinant coagulation Factor VIIa, intravenous albumin infusion, furosemide, and spironolactone. During this period, consciousness was preserved without any sign of neurological degradation. Due to the severe state of her evolution and aggravating liver disease, the patient was transferred to the Cliniques Universitaires Saint-Luc, Bruxelles, Belgium. At 10 months, the girl underwent LT from a living related donor (mother). The recipient received segments II and III from her mother’s liver (180 g, graft-to-recipient weight ratio, GRWR 2.19%). This intervention was well-tolerated, without any incidents, and there was a favorable evolution under immunosuppression therapy with tacrolimus and methylprednisolone.

Four months after LT, she was admitted to our hospital for two prolonged bacterial and Rotavirus enteritis episodes. During the 6 weeks of hospitalization, she presented hyperchloremic metabolic acidosis (blood pH 7.21, serum bicarbonate 9.6 mmol/L, blood anion gap 11 mEq/L, serum chloride 119 mEq/L), hyperkalemia (7.5 mEq/L), and hyponatremia (127 mEq/L). The urinary pH was 8.5, with a positive urinary anion gap (51.04 mmol/L) and with erythrocytes and protein absent. Plasma renin activity was low (0.74 μUI/mL), and the level of aldosterone (2.1 ng/mL) and renal ultrasound showed no alterations. These findings suggest type IV renal tubular acidosis (RTA), most likely due to a high tacrolimus serum level (34.5 ng/mL). After sodium bicarbonate supplementation (2 g/day) in association with fludrocortisone therapy (0.05 mg/kg/day) and a reduction in the tacrolimus dose, the symptoms and laboratory tests improved.

**Figure 1:**
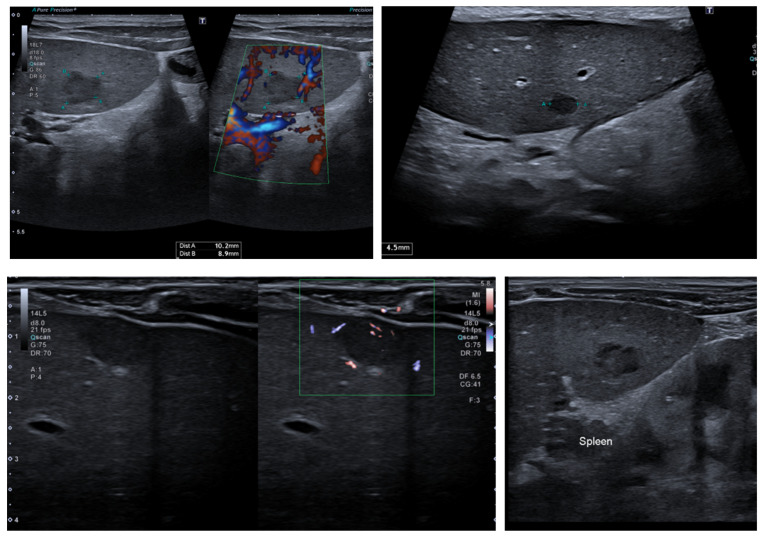
The ultrasound exam revealed three lesions on the liver and one on the spleen.

After one week at home, the patient’s clinical condition worsened, with significant weight loss (800 g) down to 7.900 g (z-score −1.93 DS), a height of 74 cm (z-score −0.77 DS), a weight for age z-score of −2.2 DS, a ponderal index (PI) of 0.79, and a total refusal of food. Thus, we started feeding her with a nasogastric tube. The neurological evaluation did not reveal any signs suggestive of the progression of the underlying disease. Also, we periodically evaluated the liver function, detecting a slight increase in transaminases and cholestasis enzymes. The abdominal ultrasound revealed four hypoechoic lesions, similar to granulomas (three on the liver and one on the spleen) (Figure 1). As tests for the Epstein-Barr virus and Cytomegalovirus’s viral load were positive, the most likely explanation was the onset of post-transplant lymphoproliferative disease (PTLD). 

At 1 year and 4 months, we referred her again to the transplant center in Bruxelles, Belgium. The reduction in the tacrolimus dose was continued, and methylprednisolone and ganciclovir therapy were started.

**Figure 2:**
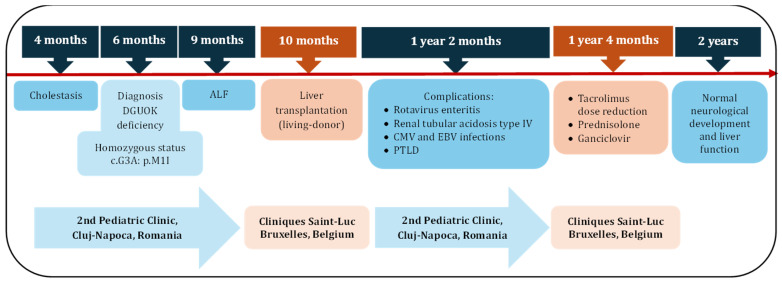
Timeline of the diagnosis, complications, and treatment in our patient with DGUOK deficiency and liver transplant (ALF, acute liver failure; CMV, Cytomegalovirus; EBV, Epstein-Barr virus; PTLD, post-transplant lymphoproliferative disorder).

Two months later, her clinical features and laboratory parameters improved considerably. The follow-up at two years of age showed normal physical and neurological development, normal laboratory parameters (AST 40 U/L, ALT 36 U/L, total bilirubin 0.44 mg/dL, conjugated bilirubin 0.15 mg/dL, albumin level 3.8 g/dL, prothrombin time 13.8s, INR 0.99, pH 7.38, serum bicarbonate 21 mmol/L, and potassium level 4 mEq/L), and no changes on the abdominal ultrasound. The level of CMV-DNA (PCR) measured negative (<88 UI/mL), and the level of EBV-DNA decreased significantly (189 UI/mL) (Figure 2). 

## 3. Discussion

DGUOK deficiency, one of the major causes of MDS, is inherited in an autosomal recessive manner. Based on clinical features, there are two forms: a multisystemic disorder with neonatal onset characterized by progressive liver disease (neonatal hepatitis, cholestasis, jaundice, and hepatomegaly) and neurological symptoms (hypotonia, nystagmus, and delayed acquisitions), and an isolated hepatic disorder (manifesting later in infancy or childhood). Patients present severe hypoglycemia with lactic acidosis, elevated ALT and AST levels, elevated GGT, and high alpha-fetoprotein (AFP). Histology can reveal cholestasis, fibrosis, giant cell hepatitis, or even cirrhosis, and the mtDNA content in liver tissue is less than 20% in matched controls [4]. Diagnosis is completed by identifying the pathogenic variants in DGUOK genes [5].

Besides jaundice, cholestasis, and hepatomegaly, liver involvement includes progressive hepatic dysfunction, leading to liver failure with ascites, coagulopathy, and edema [6]. The prognosis for DGUOK deficiency is poor; most affected children die of liver failure before 4 years of age [7]. 

Management requires a multidisciplinary team, including specialists in pediatric hepatology, neurology, general practitioners, clinical geneticists, nutrition specialists, and kinesitherapies. 

Neurologic manifestations, a prominent part of MDS, are represented in patients with DGUOK deficiency by marked hypotonia, nystagmus, and developmental delay, and inconsistently reported poor or lack of visual tracking in young infants and poor suckling reflex [6,8,9,10,11]. 

Hypotonia is evident early during infancy and tends to be profound [6]. Nystagmus develops rapidly during infancy [10,12]. In most reported cases, nystagmus is described as horizontal, yet rare descriptions of multidirectional or rotatory nystagmus have also been reported [11,12,13]. Developmental delay is evident in most patients reported in the medical literature, yet no formal, detailed descriptions of the magnitude of impairment are available [6,11,12]. However, in a long-term follow-up of 14 patients with confirmed DGUOK deficiency, only one 18-year-old patient with normal neurological development was identified. The others were reported to have varying degrees of impairment [12]. Seizures are uncommon in patients with DGUOK deficiency and are secondary to metabolic disturbances caused by decompensated liver disease [11].

Caution should be exercised when evaluating hypotonia in patients with suspected MDS, as mild hypotonia and a delay in achieving developmental milestones are not uncommon in chronically ill children or those who require prolonged hospitalizations [6]. However, these fundamentally differ from the comparably severe impairments in patients with DGUOK deficiency [6,13]. The continuous monitoring of motor development and skills, with proper intervention, if necessary, is appropriate in affected patients [7]. 

Abnormal neurological development in infants with confirmed DGUOK deficiency is associated with lower survival rates [11]. However, after long-term follow-up, it appears that nystagmus alone is associated with disease progression and lower survivability rates [12]. Hypotonia and developmental delay are not adverse predictors of survival. Thus, some authors caution against using them as exclusion criteria for LT [12].

Brain MRIs of the patients with DGUOK deficiency followed long-term do not show consistent patterns of brain abnormalities [11,12,13]. However, one report of three related patients showed a pattern of white matter lesions associated with a variant in exon 6 of the DGUOK gene [14].

Electroencephalographic (EEG) studies of patients with DGUOK deficiency are rarely reported, and there is no consistent evidence that these children have epileptic or other abnormal electrical activity [10]. There is one description of a single patient with developmental delay and a generalized slowing of electrical brain activity, yet no clinical evidence of epilepsy [12].

Our patient presented hypotonia and developmental delay. We also observed short episodes of horizontal nystagmus that, fortunately, were transient and secondary to hydro-electrolytic imbalances. Moreover, the neurological exams excluded a possible progression of the underlying disease. At the age of 1 year, the girl presented slight retardation of psychomotor development, but she sits, maintains an orthostatic position, and rises with support. The neurological reassessment at 1 year and 4 months showed acquisitions corresponding to the age of 9–10 months and a disorder of expressive language development.

Most patients with DGUOK deficiency will develop life-threatening liver failure requiring LT. The role of LT in DGUOK deficiency remains controversial, mainly due to multi-organ involvement [10,11,12,15,16,17,18,19]. Even though LT provides significant patient relief and a viable way to manage metabolic disturbances, the neurological manifestation of mtDNA depletion can appear or progress after LT [8,12]. The correct evaluation of the neurological involvement (including EEG and MRI) is essential to the indication for LT, as LT has proved beneficial in patients without significant neurological signs at the time of transplantation [16]. Even in the presence of some neurological MRI findings, without significant deterioration, LT is a good option [17]. Also, in infants with ALF, it is difficult to differentiate neurological changes and decide if they are due to DGUOK deficiency or ALF. Still, a baseline MRI may be important to evaluate brain changes before LT and to compare the evolution of brain changes after LT, if this is indicated. Moreover, the risk of other complications (pulmonary hypertension and renal impairment) after LT must be considered [12,16,19].

Starting 30 years ago, LT was indicated in patients with mitochondrial respiratory chain hepatopathies only if extrahepatic disease could confidently be excluded [18]. According to an analysis of cases published between 1995 and 2010, without a genetic diagnosis of the disease, the long-term survival post-LT was only 44%, compared to 90% in other indications [15,18]. More recently, an analysis of cases with DGUOK deficiency and LT revealed that of 20 patients transplanted before the age of 18 months, 10 (50%) died during the first 21 months after LT. Among the survivors, seven presented neurological changes after LT, ranging from mild hypotonia to severe psychomotor retardation [16]. 

The analysis of the genetic background revealed no correlation between the variants and the prognosis of the disease, but the number of reported patients is low [16]. Still, Grabhorn et al. reported that all long-term survivors of LT presented at least one variant with some residual activity of DGUOK [12]. The variant present in our patient in homozygous status (c.G3A: p.M1I) is considered a null variant, with no enzyme production or absence of functions. Even though we consider the evolution of our patient until now, almost three years after LT, as a good one, we continue to monitor her development carefully.

Our patient was transplanted using a living related donor (mother). Grabhorn et al. consider the role of living related donors in cases of DGUOK deficiency, an autosomal recessive disorder, unclear. However, 50% of liver enzyme activity is expected in a carrier, and reported cases, like our patient, benefited from this kind of donor [12]. 

So, based on these data, the LT in DGUOK deficiency should not be contraindicated in patients with or without minor neurological involvement [16]. Neither hypotonia nor psychomotor retardation can predict the clinical outcome. 

Long-term prognosis for DGUOK patients depends on neurological and other organ involvement. Even in patients with LT who have survived ALF, severe abnormal neurological findings would predict a poor prognosis, with death in 50% of patients known to be transplanted [16]. Besides the complications presented, our patient had, until now, a relatively good evolution post-LT, but there is a need for the long-term monitoring of neurological manifestations. Finally, given the genetic nature of DGUOK deficiency, it is essential to provide genetic counseling to affected patients and their families.

This report of our patient with a rare liver disease and a possible indication for LT reveals the complex management and possible difficult evolution of such cases, reflecting the need for referrals to high-volume, specialized centers for LT.

## 4. Conclusions

This case highlights the unpredictable evolution of children with LT for liver failure due to DGUOK deficiency. There are controversies regarding the indication for LT in these patients due to the possible neurological impairment. The complications of immunosuppressive therapy and the particularities of DGUOK deficiency must lead to the close monitoring of these patients to ensure the best possible long-term prognosis.

## Data Availability

The original contributions presented in the study are included in the article, further inquiries can be directed to the corresponding author.
